# Variation in Procedural Denials of Medicaid Eligibility Across States Before the COVID-19 Pandemic

**DOI:** 10.1001/jamahealthforum.2023.3892

**Published:** 2023-11-17

**Authors:** William L. Schpero, Diksha Brahmbhatt, Michael X. Liu, Chima D. Ndumele, Paula Chatterjee

**Affiliations:** 1 Division of Health Policy and Economics, Department of Population Health Sciences, Joan & Sanford I. Weill Medical College, New York, New York; 2 Center for Health Equity, Cornell University, New York, New York; 3 Department of Health Policy and Management, Yale School of Public Health, New Haven, Connecticut; 4 Department of Medicine, Perelman School of Medicine, University of Pennsylvania, Philadelphia; 5 Leonard Davis Institute of Health Economics, University of Pennsylvania, Philadelphia

## Abstract

This cross-sectional study examines denials of Medicaid and Children’s Health Insurance Program coverage due to procedural reasons.

## Introduction

Medicaid enrollment exceeded 93 million people during the COVID-19 pandemic, largely due to the Families First Coronavirus Response Act, which incentivized states to pause redeterminations of Medicaid eligibility.^1,2,3^ In April 2023, states could resume redeterminations, with estimates suggesting 17.4% of enrollees are at risk of losing coverage.^4^ Millions are projected to lose coverage for solely procedural reasons (eg, after missing a required renewal form following a change in address) despite remaining eligible.^4^ Coverage losses have been associated with reduced use of preventive care and worsened health outcomes.^5^

In this cross-sectional study, we describe the prevalence of procedural denials of coverage in Medicaid and the Children’s Health Insurance Program (CHIP) across states in 2019, before the pause on redeterminations. We also evaluate the association between rates of procedural denials and states’ renewal processes to determine whether states’ upstream operational investments may protect against high denial rates.

## Methods

Data were obtained via a Freedom of Information Act Request for the Centers for Medicare & Medicaid Services (CMS) Medicaid and CHIP Eligibility and Enrollment Performance Indicators data set for calendar year 2019. We calculated the rate of procedural denials in each state as the proportion of total eligibility determinations in which individuals were deemed ineligible for Medicaid or CHIP because they “failed to complete or return renewal forms or other required documentation, or … were lost to follow up.”^6^ We also calculated the proportion of eligibility determinations for renewals vs new applications. We limited analysis to states with at least 6 months of data for these 2 measures in 2019 that CMS had not flagged for data quality concerns. This study followed the STROBE reporting guideline and was deemed not human participants’ research by the institutional review board at Weill Cornell Medical College.

We visualized mean rates of procedural denials across states for Medicaid and CHIP. We then used scatterplots and Wilcoxon rank sum tests to examine the association between procedural denial rates and 2 measures of states’ renewal processes in 2019—rates of automated renewals and use of renewal forms prepopulated with updated enrollee information—obtained from KFF.^7^ Analyses were performed using Stata, version 16 (StataCorp). With 2-sided testing, *P* < .05 was considered statistically significant. eMethods in Supplement 1 provides further details.

## Results

**Figure 1.**
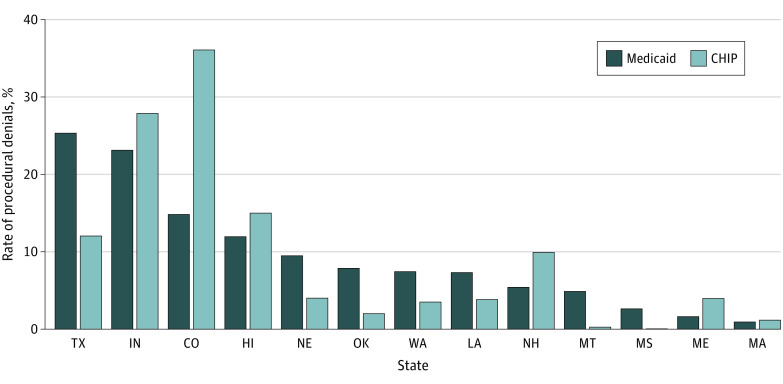
Procedural Denial Rates in Medicaid and Children’s Health Insurance Program (CHIP) Across States, 2019 State rank ordered by rate of procedural denials in Medicaid.

Only 13 states (comprising 20.0% of enrollees nationally) had sufficient data quality for inclusion. Among those states, most eligibility determinations involved renewals rather than new applications (median, 71.4% [IQR, 50.9%-83.1%]). Rates of procedural denials in Medicaid ranged from 0.9% to 25.4% (median, 7.4% [IQR, 4.9%-11.9%]); rates in CHIP ranged from 0.04% to 36.1% (median, 4.0% [IQR, 2.0%-12.0%]) (Figure 1).

**Figure 2.**
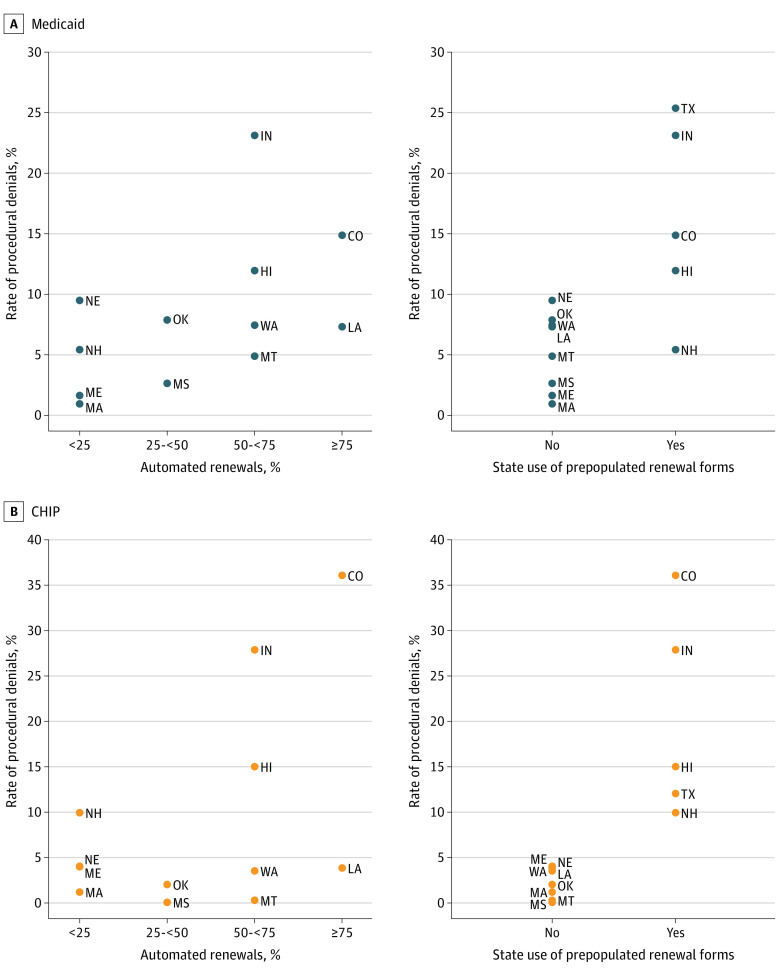
Association Between States’ Procedural Denial Rates in Medicaid and Children’s Health Insurance Program (CHIP) and Renewal Processes, 2019 Automated renewals data not available for Texas.

Median rate of procedural denials for Medicaid was 11.1% (IQR, 7.3%-14.9%) in states that automated 75% or more of renewals vs 3.5% (IQR, 1.3%-7.4%) in states that automated less than 25% of renewals, but the difference was not statistically significant (*P* = .16) (Figure 2A). Rates were significantly higher in states that used prepopulated and updated renewal forms (median, 14.9% [IRQ, 11.9%-23.2%]) compared with rates in states that did not (median, 6.1% [IQR, 2.1%-7.7%]) (*P* = .02). Similar associations were evident in CHIP (Figure 2B).

## Discussion

We found few states reported high-quality data on procedural denials before the COVID-19 pandemic. Among reporting states, there was wide variation; in certain states, more than 1 in 4 Medicaid determinations and 1 in 3 CHIP determinations resulted in ineligibility solely for procedural reasons; in others, procedural denials were close to 0. Procedural denials were higher in some states that had invested in processes designed to improve the renewal process, suggesting that these processes alone may be insufficient to protect against high denial rates.

This study was limited to a small subset of states due to data quality concerns in the remainder. We were also unable to separately examine denials for renewals vs new applications. These limitations highlight the need for CMS to work with states to improve quality and availability of data on determinations—and procedural denials, specifically—to monitor for potentially inappropriate denials of coverage and to enable corrective action, if necessary.

## Notes

Supplement 1.
eMethods

Supplement 2.
Data Sharing Statement
