# Supplementary material for: Variation in Procedural Denials of Medicaid Eligibility Across States Before the COVID-19 Pandemic
Source: JAMA Health Forum. 2023 Nov 17;4(11):e233892. doi: 10.1001/jamahealthforum.2023.3892 (PMC10656645; doi:10.1001/jamahealthforum.2023.3892)
Supplement: Supplement 1. — eMethods [file jamahealthforum-e233892-s001.pdf]

Supplementary Online Content

Schpero WL, Brahmbhatt D, Liu MX, Ndumele CD, Chatterjee P. Variation in procedural denials of Medicaid eligibility across states before the COVID-19 pandemic. *JAMA Health Forum*. 2023;4(11):e233892. doi:10.1001/jamahealthforum.2023.3892

eMethods

This supplementary material has been provided by the authors to give readers additional information about their work.

© 2023 Schpero WL et al. *JAMA Health Forum.*

eMethods

Rates of procedural denials were derived from the Centers for Medicare and Medicaid Services (CMS) Medicaid and Children's Health Insurance Program (CHIP) Eligibility and Enrollment Performance Indicators dataset, which was obtained via a Freedom of Information Act (FOIA) request. The FOIA request was filed on March 10, 2022; data were received in response to the request on May 13, 2022. The dataset reflects monthly reports from states to CMS on key operational measures, including applications, renewals, eligibility determinations, and enrollment.

Rates of procedural denials were generated using the following variables:

Indicator Number	Title	Description
9a	Total Medicaid Eligible	Total number of individuals determined eligible for Medicaid (i.e. funded under title XIX of the Social Security Act) under either MAGI or non-MAGI rules during the calendar month. This count should include determinations following an initial application as well as all redeterminations (triggered by either the annual renewal process or other change in circumstances). Individuals determined eligible for CHIP (i.e. funded under Title XXI of the Social Security Act, including through MCHIP programs) are not included in this indicator.
10a	Total Medicaid Ineligible	Total number of individuals determined ineligible for Medicaid (i.e. funded under title XIX of the Social Security Act) under either MAGI or non-MAGI rules during the calendar month. This number should include determinations following an initial application as well as all redeterminations (triggered by either the annual renewal process or other change in circumstances). Include final determinations made by any state agency, including the Medicaid agency, a separate CHIP agency, and an SBM. Include individuals determined ineligible by a state agency after their account was assessed and transferred from the FFM. Do not include final eligibility determinations made by the FFM.
10c	Medicaid Determination – Eligibility Cannot be Established	Total number of individuals determined ineligible for Medicaid (i.e. funded under title XIX of the Social Security Act) under either MAGI or non-MAGI rules during the calendar month because they failed to complete or return renewal forms or other required documentation, or who were lost to follow up.

© 2023 Schpero WL et al. *JAMA Health Forum*.

9j	Total CHIP Eligible	Total number of individuals determined eligible for CHIP (i.e. funded under Title XXI of the Social Security Act, including through MCHIP programs) during the calendar month. This number should include determinations following an initial application as well as all redeterminations (triggered by either the annual renewal process or other change in circumstances).
10g	Total CHIP Ineligible	Total number of individuals determined ineligible for CHIP ((i.e. funded under Title XXI of the Social Security Act, including through MCHIP programs)) during the calendar month. This number should include determinations following an initial application as well as all redeterminations (triggered by either the annual renewal process or other change in circumstances). Include final determinations made by any state agency, including the Medicaid agency, a separate CHIP agency, and an SBM. Include individuals determined ineligible by a state agency after their account was assessed and transferred from the FFM. Do not include final eligibility determinations made by the FFM.
10i	CHIP Determination – Eligibility Cannot be Established	Total number of individuals determined ineligible for CHIP (i.e. funded under Title XXI of the Social Security Act, including through MCHIP programs) during the calendar month because they failed to complete or return renewal forms or other required documentation, or who were lost to follow up.

For Medicaid, rates of procedural denials were calculated as 100*(10c/(9a+10a)). For CHIP, rates of procedural denials were calculated as 100*(10i/(9j+10g)).

The Performance Indicators dataset flags variables by state and month if there is a known data quality issue; any state-month variable with a data quality flag was recoded to missing. Most of the reported issues involved cases where data submitted by states for a given indicator and time period were missing, partially complete, or incorrect (i.e., the state did not follow the specifications provided by CMS). Only states with at least 6 months of non-missing data in 2019 for all variables used to construct the denials measures, as well as the measure of the proportion of eligibility determinations (excluding procedurally denials) that were for renewals versus new applications, were included in the sample.

© 2023 Schpero WL et al. *JAMA Health Forum.*
