# Supplementary material for: Variation in Procedural Denials of Medicaid Eligibility Across States Before the COVID-19 Pandemic
Source: JAMA Health Forum. 2023 Nov 17;4(11):e233892. doi: 10.1001/jamahealthforum.2023.3892 (PMC10656645; doi:10.1001/jamahealthforum.2023.3892)
Supplement: Supplement 2. — Data Sharing Statement [file jamahealthforum-e233892-s002.pdf]

Data Sharing Statement

Schpero. Variation in Procedural Denials of Medicaid Eligibility Across States Before the COVID-19 Pandemic. *JAMA Health Forum*. Published November 17, 2023. doi:10.1001/jamahealthforum.2023.3892

Data
Data available: Yes
Data types: Data (not involving human participants), Data dictionary
How to access data: Data are available upon request from the corresponding author at wls4001@med.cornell.edu.
When available: With publication

Supporting Documents
Document types: None

Additional Information
Who can access the data: Data will be made available to anyone requesting the data.
Types of analyses: Data will be made available for any purpose.
Mechanisms of data availability: Data will be made available to anyone requesting the data.
